# Is manure an alternative to topsoil in road embankment restoration?

**DOI:** 10.1371/journal.pone.0174622

**Published:** 2017-03-27

**Authors:** Begoña Peco, Desirée Rivera, Pablo García-Palacios, Berta M. Jauregui

**Affiliations:** 1 Departamento de Ecología, Universidad Autónoma de Madrid, Madrid, Spain; 2 Área de Biodiversidad y Conservación, Departamento de Biología y Geología, Universidad Rey Juan Carlos, Móstoles, Madrid; 3 Dirección Técnica, Obrascón Huarte Lain, S.A. (OHL), Madrid, Spain; Estacion Experimental de Zonas Aridas, SPAIN

## Abstract

One of the main steps in road and railway embankment restoration is the spreading of previously removed topsoil, which provides an input of seeds, organic matter and microorganisms and encourages the establishment of a vegetation cover, essential to stabilise the embankment and blend it with the landscape. However, topsoil is a scarce resource, prompting the search for economic alternatives with similar results. The present study compares the results of spreading topsoil with an organic amendment (manure) for the soil's physico-chemical properties, erosion resistance and microbial activity, floristic richness and composition, and bare soil cover. For this purpose, experimental plots with three treatments (Control, Topsoil and Manure) were maintained on a recently built embankment in Central Spain for 20 months. Manure was found to be an effective alternative to topsoil for the improvement of soil fertility (organic matter content and total nitrogen). The two types of organic amendment produced similar reductions in bare soil cover and erosion rates. However, plots with topsoil showed greater soil respiration and species richness and a different floristic composition in comparison to those treated with manure, which was closer to control plots. These results suggest that manure can be used to replace topsoil to enhance embankment stability during the early stages of restoration. However, if the aim of the restoration process is to promote plant diversity, topsoil is recommended.

## Introduction

Linear infrastructure—motorways and railway lines—has undergone a major expansion in recent years due to population growth and increased mobility. Road easements cover 1% of the land area in most developed countries [1]. An estimated 1.5% of the European Union is covered by motorways and 5% by railway lines [2]. This area is expected to increase in the coming years.

Steep embankments and large areas lacking vegetation are generated during the construction of linear infrastructures [3–5]. Embankments are artificial structures built where the infrastructure platform has to cross hills. They generally have steep slopes, nutrient-poor soils and little vegetation cover [6, 7], making them vulnerable to deleterious erosion and consequently high environmental and financial costs [8, 9]. These features and the lack of a natural reference ecosystem hinder their restoration, and they often require major investment by the authorities that exploit the infrastructure [10]. Restoration of the plant cover on embankments is indispensable during the initial stages immediately after a road or railway track is built in order to prevent soil erosion, ensure the consolidation of these structures and integrate them into the landscape [11]. This in turn facilitates connectivity between habitats, the reestablishment of flows, functions and processes, and the overall restoration of the disturbed land [12].

The re-establishment of the vegetation cover is usually accelerated by hydroseeding [5, 13–18]. Before these techniques are applied, plant colonisation and growth is facilitated with a layer of topsoil spread across the surface to be restored [19–21]. Ideally, this fertile soil should be taken from the top 30 cm of the material removed prior to the earthworks. It is stockpiled in pyramids of varying height for different periods, depending on the civil engineering process [22, 23].

Topsoil is a valuable resource for the restoration of degraded zones due to its high contents in native seeds [21, 24, 25], nutrients and microorganisms [26]. Its contribution to the establishment and stabilization of the vegetation cover in disturbed areas has been proven in studies of roadside slope [6, 20–23,27] and mine restoration [24,28–31]. However, there is often a shortage of topsoil in civil engineering works because the area to be restored is usually larger than the area of the infrastructure platform *per se*. For this reason, when not enough fertile soil is available to restore the entire disturbed area, poor quality soil from deeper layers is employed, compromising the success of the restoration process[31]. Although other sources are available, few studies have compared the effectiveness of topsoil with other organic amendments in the restoration of the vegetation cover and the soil's former physico-chemical properties on road embankments.

Farm soil fertility is improved by applying fertilizers that changes its physico-chemical properties. Fertilizers are complex, specific and expensive, offset by the higher crop yield. Animal manure also improves the physical, chemical and biological properties of soil [32–37] and, in contrast to synthetic fertilisers, is easy to obtain at low cost from the increasing numbers of intensive livestock farms. Manure improves soil fertility and promotes plant growth by adding organic matter and nutrients such as nitrogen, phosphorus and potassium, as well as through increased soil pH [36, 38]. Organic matter has a positive effect on the soil water holding capacity, and thus increases the amount of water available for plants [39]. In addition to its positive effect on different pools and flows of carbon and nutrients, manure also increases soil heterotrophic respiration via the microbial biomass [40]. Manure thus has positive effects on plant performance and soil functioning.

In recent years, new applications for manure have been tested for the dual purpose of employing this organic nitrogen-rich resource and reducing surplus waste from livestock farms whose management requires non-amortizable investment by the farmer. Examples include the use of manure to improve semiarid soils [41] and former mines [42].

Fertilizers have been used in the revegetation of road embankments to accelerate the growth of the vegetation cover [43–45], but no cases of manure used for the same purpose have been published in scientific literature. Agroecosystem soils amended with manure contain greater biological activity and more organic matter in the long term than fertilizer-treated soil [35], supporting its use as an organic amendment to improve the results of restoration in harsh, nutrient-poor situations such as embankments.

The present study aimed to compare the effects of organic amendments (horse manure) and topsoil on soil physico-chemical properties (organic matter, total nitrogen, assimilable phosphorus and potassium, texture, soil water holding capacity, pH and compaction), soil microbial activity, erosion resistance, bare soil cover, plant species richness and species composition on linear transport infrastructure embankments in the centre of the Iberian Peninsula.

Our hypotheses were: (H1) topsoil increases vegetation cover and species richness, reduces bare soil cover and erosion, and improves soil fertility, and (H2) manure yields similar results to those of topsoil in terms of bare soil cover and erosion reduction, increased concentration of organic matter and promotion of biological activity in the soil. Finally, the advantages and disadvantages of manure as an alternative to topsoil in embankment restoration are discussed.

## Material and methods

### Study area

The study area was east of Madrid (Spain) in Dehesa de Mari Martín, Navalcarnero municipality (40° 18’ N; 3° 58’ W). The experimental zone was a railway easement under construction between two towns, Móstoles and Navalcarnero. The area lies on sandstone and has a semiarid Mediterranean climate, with cold winters and hot, dry summers. The mean minimum and maximum temperatures are 9 and 20°C, respectively, with 450 mm mean annual precipitation, mainly falling in autumn and spring.

### Experimental design

In December 2009, we selected a recently shaped north-facing embankment beside a railway line on a 14° slope. Prior to the treatments, the slope was cleared mechanically to remove the top 10 cm of the soil surface in order to ensure similar initial conditions in each plot. In October 2010, we staked out fifteen contiguous 4 x 9 m experimental plots, set in five blocks. The longer dimension of the plots follows the line of maximum slope. Three treatments were applied to the plots: Topsoil, Manure and Control. In the Topsoil treatment, fertile soil was spread mechanically to a uniform depth of 10 cm. This topsoil had been collected in December 2009 when the upper 30 cm of the land near the experimental embankment was removed. This material was stored for 10 months in 2 m high x 1.5 m wide pyramidal stockpiles, the common practice in this type of earthwork. The Manure treatment consisted of manually spreading horse dung at a ratio of 2 kg dry weight/m^2^ across five of the experimental plots. Horse manure was chosen because its high organic matter and nitrogen content [46]. The manure was collected from a stud farm in the same municipality. The manure amendment used in the treatments consisted of a 3:1 mixture of horse dung and pine shavings (hereafter Manure), left in the open air for 8 months (February to October). The horses were fed a mixture of straw, hay and high-quality grainfeed. The Control plots received no treatment at all.

### Soil physico-chemical properties

In October 2010, following the treatments, the initial physico-chemical properties of the soil were tested using a composite soil sample per plot, collected using at least eight 4 cm diameter x 10 cm deep soil cores. These samples were air dried for 10 days and passed through a 2 mm mesh sieve. We then analysed the content in organic matter [47], total nitrogen (N; Kjeldahl), phosphorus (P) and assimilable potassium (K; EDTA acetate extraction; [48], percentages of sand, silt and clay [49] and water holding capacity [50]. Soil pH was measured in a 1:2.5 soil/water solution.

### Soil microbial respiration

Basal soil respiration or CO_2_ production rate (g CO_2_ m^-2^ h^-1^1) is widely used as an index for estimated soil microbial activity, given that the majority corresponds to the heterotrophic respiration of microorganisms and not the autotrophic respiration of plant roots [51, 52]. This parameter was measured monthly for one year (December 2010 to November 2011) between 10 am and 12 pm to avoid heavy diurnal fluctuations. Soil respiration was measured at three points (upper, middle and low zone) in each plot using a PVC cylinder, then averaging the data for each plot and date. For this purpose, we used an Environmental CO2 Gas Monitor (EGM-4) fitted with a Closed System Soil Respiration monitor (SRC). The sparse vegetation at the measurement points was removed a week before each measurement to prevent interference by the autotrophic respiration of the roots of freshly removed plants.

### Soil compaction and erosion

In March 2011 we measured soil compaction in the field using an IB penetrometer (Eijkelkamp Agrisearch Equipment BV, Giesbeek, Netherlands) at five random points in upper and lower zones of each plot. To determine the relative amount of erosion on the slope, we measured the width (W) and depth (D) of all rills found in each plot in October 2011 and November 2013 along two 2 m transects running perpendicular to the maximum slope, set 1 m from the edges of the plots, one in the upper part and the other in the lower part of the embankment. For each plot, zone and date we noted two erosion indices: number of rills (R) and rill size, as ΣW x ΣD of all rills intercepted by the transect.

### Bare soil cover, plant species richness and floristic composition

In May 2011 and 2012, the optimum phenological period for the study of herbaceous annual communities on semi-arid embankments, we visually assessed the area of bare soil cover and every species cover in six 0.5 x 0.5 m quadrats (Three quadrats were located in the upper and three in the lower parts of the plot, distant 1 m between them and from the border of the plot). We also calculate species richness per quadrat.

### Data analysis

The effects of the treatments on soil compaction and physico-chemical properties were analysed with ANOVAs. The effect of the treatments on soil respiration, bare soil cover, soil compaction, erosion and species richness were analysed with repeated measures ANOVAs. Time was included as a between factor, while treatment (Topsoil, Manure and Control) and topographic position (upper and lower) were used as within-subject factors. The dependent variables were log-transformed where necessary to fulfil the assumption of normality. SPSS 15.0 (SPSS Inc., Chicago, IL, USA) was used for these analyses.

The response of floristic composition to the treatments over time was tested using Redundancy analysis (RDA) and Principal Response Curves (PRC) [53], starting with an RDA and inserting the treatments and slope zones as environmental variables and years as repeated measures. The significance of the treatments for floristic composition was tested with a Monte Carlo permutation test (499 permutations) and automatic selection. A PRC was then used to detect the temporal change in the floristic composition of the samples for each treatment. In this case, slope position was not used as an environmental variable due to its non-significance in the previous RDA analysis. PRC is a particular kind of redundancy analysis (RDA), in which interaction between treatments and years were the explanatory variables and years were the covariables [54]. The significance of the first axis of this RDA was checked also with a Monte Carlo permutations test of 499 permutations on the plots (n = 49) but not the two years [54]. The result was a diagram with the first principal component of the variance explained by the treatments on the y-axis and time in the x-axis. The control treatment was used as the zero base line and the treatment effects were represented by the deviation of each treatment line from this base line. Time, treatments and slope zones were coded as dummy variables and species values were centred. CANOCO 4.5 [55] was used for the RDA and PRC, and CANODRAW for Windows 4.12 [56] for the graphics.

### Ethics statement

Prior to the field studies, we obtained all the permits for the works. The Site Manager of Obrascón Huarte Lain S.A. and Madrid Regional Government were informed, since experimental plots were located on an embankment under construction. No protected species were sampled during the study.

## Results

### Soil physico-chemical properties

Soils in Manure plots had a higher P, K and water holding capacity than the Topsoil and Control plots (Table 1). The organic matter and N content in the Manure plots was as high as the Topsoil plots. pH was the only parameter that differed significantly between the three treatments, increasing in the following order: Topsoil, Manure and Control. The soil in the Control plots showed no significant difference from the Manure plots in the measured physical parameters. However, Topsoil plots had a smaller proportion of clay and more silt than the other two treatment plots (Table 1)

**Table 1:** Soil physico-chemical parameters (mean ± EE) for each treatment. Differences between treatments (g.l = 2; n = 15) are shown with letters. WC: Water holding capacity, OM: organic matter, P: assimilable Phosphorous, K: assimilable Potassium, N: Total Nitrogen. Different letters indicate significant differences between treatments (Post hock Turkey test, *P*<0.05).

Parameter	Control	Topsoil	Manure	F	*P*
WC (g/kg)	58 ± 6.25^a^	52.7 ± 1.82^a^	69.4 ± 9.13^b^	8.65	0.05
OM (g/kg)	1.2 ± 0.86^a^	10.0 ± 1.02^b^	9.7 ± 3.15^b^	32.46	<0.001
P (mg/100g)	0.2 ± 0.04^a^	0.2 ± 0.08^a^	1.1 ± 0.18^b^	88.19	<0.001
N (g/kg)	0.2 ± 0.03^a^	0.7 ± 0.08^b^	0.6 ± 0.11^b^	50.81	<0.001
K (mg/100g)	6.2 ± 0.64^a^	5.9 ± 0.38^a^	22.5 ± 8.67^b^	17.90	<0.001
pH	7.4 ± 0.29^c^	5.3 ± 0.04^a^	6.6 ± 0.62^b^	35.31	<0.001
Clay (g/kg)	169.3 ± 13.03^b^	99.7 ± 4.63^a^	177.0 ± 17.41^b^	55.02	<0.001
Sand (g/kg)	722.3 ± 22.61	721.7 ± 25.18	698.1 ± 16.59	2.02	>0.05
Silt (g/kg)	108.4 ± 12.63^a^	178.6 ± 25.78^b^	124.9 ± 14.27^a^	19.62	<0.001

### Soil microbial respiration

**Fig 1:**
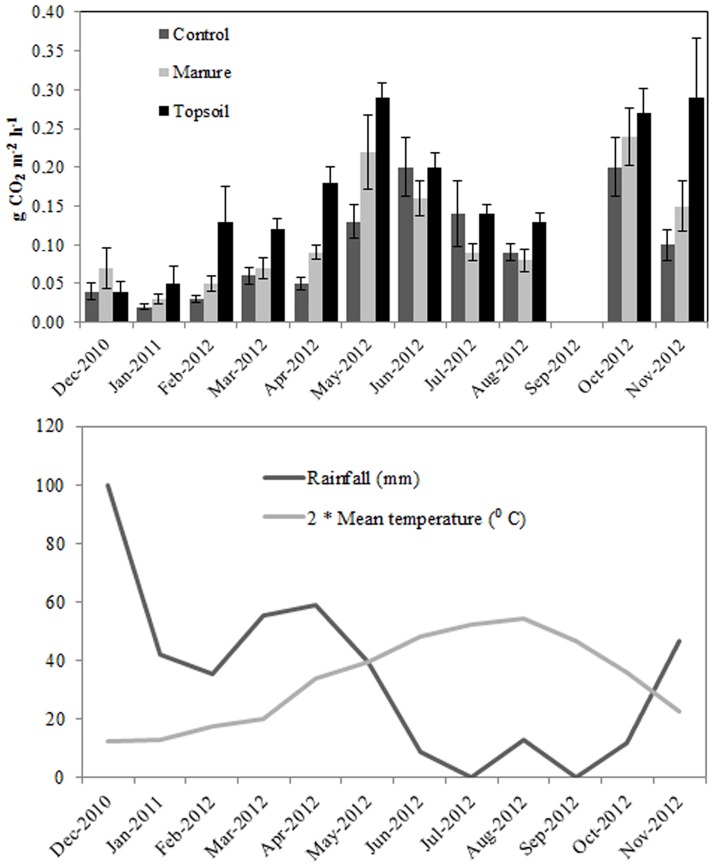
A) Graph representation of the period December 2010 to November 2011 showing mean monthly CO_2_ production rate (g CO_2_ m^-2^ h^-1^) in the experimental plots by each treatment: Control, Manure and Topsoil. The error bars show the standard error. B) Ombrothermic diagram of the study area for the same period.

Soil respiration varied through the year (F = 64.9, *P* <0.001). The lowest soil respiration data coincided with the months with the lowest rainfall and temperatures, while the peak matched the period with the highest moisture and higher temperature conditions (Fig 1). The repeated measures analysis showed a significant effect of the treatment (F = 9.03; *P* <0.01), with a significantly higher rate of CO_2_ production generally found in the Topsoil plots than the Manured and Control plots. Neither position nor interaction with the treatment were significant (*P*> 0.05 in both cases).

### Soil compaction and erosion

**Fig 2:**
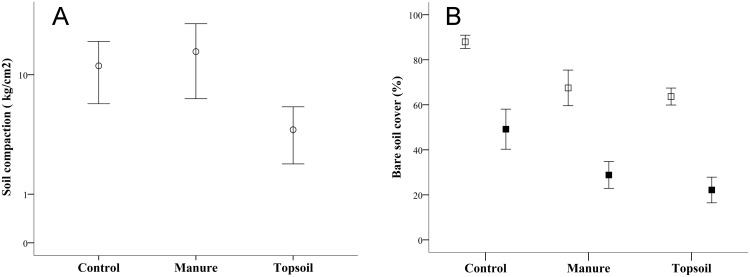
A) Soil compaction measured in March 2011 and B) Bare soil cover in the two sampling seasons (May 2011: open quadrats) and May 2012 (black quadrats) in experimental plots (Control, Manure and Topsoil). Error bars represent 95% confidence intervals

A significant effect of the treatment was found in the case of compaction (F = 8.66, *P* <0.001), as Topsoil plots were found to be less compact than the Control and Manure plots (Fig 2a). However, there was no significant effect of zone or interaction on this parameter (F = 1.42, *P* = 0.24 and F = 1.61, *P* = 0.22, respectively).

**Fig 3:**
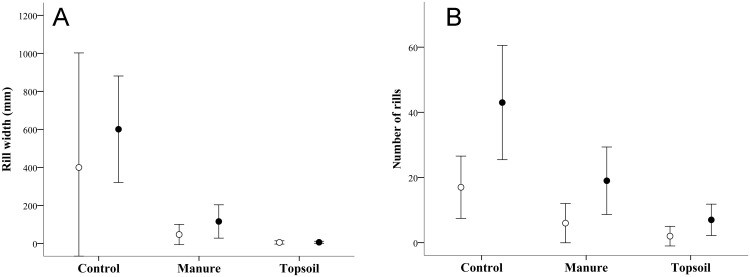
A) Mean number of rills for the two experimental years and B) Accumulated rill width in 2 m transects on experimental plots in the upper (open circles) and lower (black circles) parts of the embankment (Control, Manure and Topsoil). Error bars represent 95% confidence intervals

The type of treatment had a significant effect on both the number and size of the rills (F = 14.06, *P* <0.001 and F = 13.25, *P* <0.001, respectively). Both of these erosion parameters were highest in Control plots and slightly higher in Manure than Topsoil plots (Fig 3a and 3b). There was also a significant effect of position area (F = 14.18, *P* <0.01 and F = 6.84, *P* <0.01, respectively), with higher values of both erosion indicators in the lower zones of the embankment (Fig 3a and 3b). None of these parameters showed significant effects of time (F = 1.69, *P* = 0.20 and F = 0.01, *P* = 0.92, respectively) or time × treatment interaction (F = 0.82, *P* = 0.43 and F = 0.44, *P* = 0.65, respectively).

### Bare soil cover, plant species richness and floristic composition

**Fig 4:**
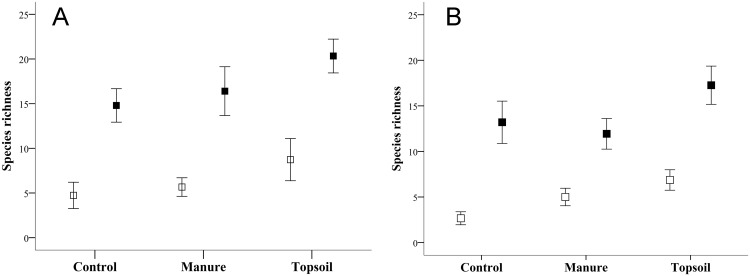
Species richness for (A) upper slope zones and (B) lower slope zones in the two sampling seasons (May 2011: open quadrats) and May 2012 (black quadrats) in experimental plots (Control, Manure and Topsoil). Error bars represent 95% confidence intervals.

**Fig 5:**
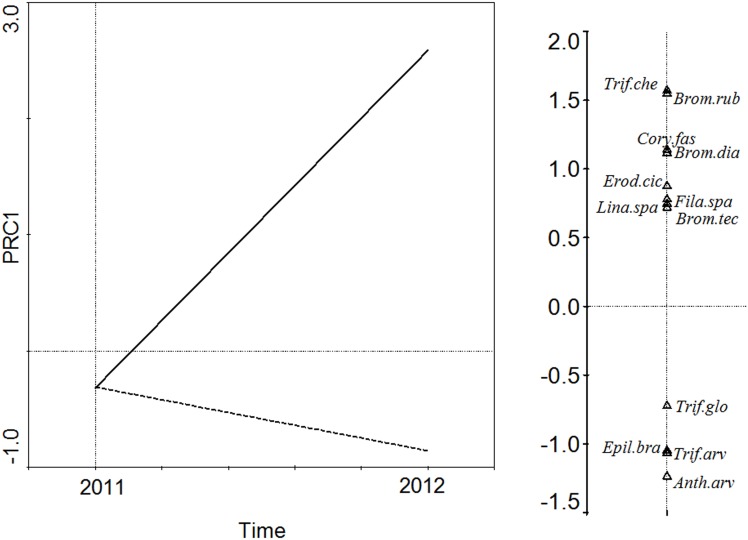
Species composition response to treatments. Y-axis in Principal Response Curves PRC diagram: first component of variation explained by differences in treatment over time. X-axis: control treatments. Dotted line: manure treatment. Solid line: topsoil treatment. The weighting of each species on the principal axis (right of PRC diagram) represents affinity of each species to treatments. Species with positive weighting in PRC1 are abundant in Topsoil plots while species with negative weighting are abundant in Manure plots. Only species showing fits greater than 5 are shown. **Anth.arv,** *Anthemis arvensis*; **Brom.rub,** *Bromus rubens*; **Brom.dia,** *Bromus diandrus*; **Brom.tec,** *Bromus tectorum*; **Cory. fas,** *Corynephorus fasciculatus*; **Epil. Bra,** *Epilobium brachicarpum;* **Fila spa,** *Filago spatulata;* **Lina.spa,** *Linaria spartea*; **Trif.arv.,** *Trifolium arvense*; **Trif.che,** *Trifolium cherleri*; **Trif.glo,** *Trifolium glomeratum*.

Bare soil cover was higher in the Control than the Topsoil and Manure treatments, with no difference between the latter two (F = 35.67, *P* <0.001, Fig 2b). This parameter decreased with time (F = 310.81, *P* <0.001), with no significant effect of location or interaction with treatment (F = 2.78, *P* = 0.10 and F = 1.92, *P* = 0.15, respectively). Species richness was greater in the Topsoil than the Manure and Control treatments during the two sampling years (F = 32.39, *P* <0.001, Fig 4a and 4b). It was also higher in the upper areas of the embankment (F = 17.92, *P* <0.001), and increased with time (F = 60, *P* <0.001). There was no significant effect of interaction (F = 0.39, *P* = 0.87). The automatic selection of the first RDA shows that floristic composition differed between the Topsoil treatment and the other two (Control and Manure), with no significant differences between latter two (F = 4.32, *P* = 0.07). Slope position was also non-significant (F = 1.53, *P* = 0.34). Only the first axis of the PRC was significant (F = 22.23, *P* = 0.02). The PRC diagram (Fig 5) shows a year-by-year comparison between the treatments and the designated “control” (horizontal axis). The PRC confirmed the homogeneity of the plots in Spring 2011. Only the Topsoil plots differed from the Control and Manure plots after two years of the experiment.

## Discussion

### Soil fertility and microbial activity

The results of this study show that horse manure can be a useful alternative to spread topsoil if the aim is to improve variables related to soil fertility, organic matter content and total N. This improvement is indispensable to overcome the abiotic filter that influences the establishment of plant communities on embankments in Mediterranean environments, where soils are usually poor and rainfall is low [4]. Several authors have discussed the difficulty of ensuring stable vegetation cover on embankments due to poor soil quality, amongst other hindrances, [5, 21–23, 57], making the input of nutrient-rich substrates a good option to enhance restoration. In our study both topsoil and manure increased the organic matter and N concentration in soils compared with the control plots, but spread manure contributed to a higher concentration of soil nutrients that are essential for plant growth (P and K), than areas amended with topsoil. Several authors have noted that manure is a major source of soil nutrients in different ecosystems [36, 38, 42, 49, 58, 59]. Similarly, the present study shows that topsoil and manure amendments can improve the soil fertility of embankment, despite the fact that the previous levels of organic matter and N in these soils were lower than those in other similar restoration projects [5, 21, 22, 57, 60]. Thus, manure is a viable alternative to topsoil in linear infrastructure restoration projects aimed at improving soil fertility on embankments in Mediterranean environments. Importantly, manure is easier to obtain and handle than topsoil.

Several studies have shown that biochemical indices such as soil respiration and microbial biomass are sensitive indicators of changes in soil organic matter quality and nutrient cycling following the implementation of new management strategies [61–63]. We found that amendments rich in organic matter increased microbial activity, confirming our hypothesis, and topsoil achieved better results in this respect than manure. Organic matter is one of the most important sources of energy and nutrients for microbial growth. Excess nutrient availability and exogenous labile carbon sources, especially beneath topsoil, can stimulate heterotrophic microorganism activity [63], presumably due to shifts in the microbial community towards species with high turnover rates or greater microbial biomass cycling. We propose the following mechanisms to explain the greater increase in soil respiration found in the topsoil compared to the manure plots: i) higher proportion of labile C in the topsoil, or ii) higher C:N ratio of manure, which may reduce microbial growth and enzyme production by hindering the supply of microbial N [64]. Further research to clarify these potential hypotheses would be needed in future, since our study did not measure labile C or C/N ratio.

We also found a higher rate of soil respiration in all the experimental plots, coinciding with the months of peak soil moisture and temperature, which favour microbial activity [65], especially in disturbed soil [66, 67]. Monthly soil respiration data in plots with spread topsoil or manure (0.04–0.29 and 0.03–0.24 g CO_2_ m^-2^ h^-1^) were similar to those found in temperate grassland (0.05 g CO_2_ m^-2^ h^-1^) [68], but lower than those measured in Mediterranean forests (0.268 and 0.887 g CO_2_ m^-2^ h^-1^) [69] since tree roots, non-existent in the present study, greatly increase the respiration rate [70, 71].

### Soil erosion and compaction

Spread topsoil and manure had an equivalent detrimental effect on slope erosion measured by the number and size of rills. Both indicators were higher in the lower than the upper embankment zones, possibly due to the cumulative effect related to the slope [72]. The high soil organic matter content prompted by both manure and topsoil may have contributed to the formation of stable aggregates in the soil [35, 73], reducing erosion rates. However, soil compaction was higher in plots with manure than with topsoil. This is a congruous result considering that the relatively soft topsoil layer was 10 cm deep, while the manure was spread in a layer measuring less than 3 cm, and consequently, soil physical properties did not differ between Manure and Control treatments. For the same reason, the clay content was higher in the plots restored with manure than with topsoil, which may have contributed to the compaction found beneath this organic amendment, given that finer soil textures are associated with more compact soils. Previous studies have found that soil compaction hinders the establishment of soil microbiota [74], which in turn directly affects the metabolism of the soil ecosystem and explains the poorer response of soil respiration to the manure vs. the topsoil treatment in this study.

### Bare soil, plant richness and community composition

Spread topsoil and manure produced a similar reduction in bare soil cover associated with erosion [4, 75]. This result is consistent with those found by other authors on road embankments where topsoil had been spread [21]. Both treatments seem to have facilitated colonization by herbs from first months after the slope had been formed. This result is important as it suggests that manure can replace the complicated and expensive topsoil handling process when slopes have to be revegetated quickly immediately after their construction.

On the other hand, plots with spread topsoil had greater species richness and a different floristic composition from those with manure, which resembled the control plots in this aspect. This result is possibly associated with the greater viable seed content and species richness of the seed banks in the plots covered with topsoil than those with manure quantified by Rivera [76], in the same experimental plots. This author found that the viable seed density in seed banks in zones treated with topsoil was around 5100 viable seeds m^-2^. After 10 months' stockpiling followed by spreading on the experimental plots, the number of viable seeds declined to 3501 viable seeds m^-2^ and 28 species, while the seed banks in plots with manure contained 3422 viable seeds m^-2^ and 14 species. It should be noted that the source of the manure employed in this experiment was an intensively managed stud where the horses were not fed natural grass. Manure from farms with extensive summer grazing would have contributed a larger number of seeds and might therefore be more useful for restoring the vegetation cover [77].

## Conclusions

Topsoil is a highly valuable resource for restoring degraded land, although its availability is often limited because the surface area to be restored is usually larger than that of the road or railway easement from where it is removed. Low cost, widely available organic amendments such as manure seem to be a viable alternative, depending on the objectives of the restoration project. The use of both topsoil and manure is recommended to accelerate restoration and revegetation of slopes during the early stages after linear infrastructure construction, when physical stabilization is a priority given the high erosion rates found on recently build embankments. Furthermore, topsoil is recommended when restoration is aimed at increasing or maintaining the diversity of the local vegetation.

## Supporting information

S1 File Dataset used for the analysis. (RAR)
